# Information leakage in enzyme-substrate prediction

**DOI:** 10.1093/bioinformatics/btag733

**Published:** 2026-10-01

**Authors:** Vahid Atabaigi Elmi, Roman Joeres, Olga V Kalinina

**Affiliations:** Drug Bioinformatics, Helmholtz Institute for Pharmaceutical Research Saarland, Saarbruecken, Saarland 66123, Germany; Centre for Bioinformatics, Saarland University, Saarbruecken, Saarland 66123, Germany; Department of Chemistry and Molecular Biology, University of Gothenburg, Gothenburg 41390, Sweden; Wallenberg Centre for Molecular and Translational Medicine, University of Gothenburg, Gothenburg 41390, Sweden; Drug Bioinformatics, Helmholtz Institute for Pharmaceutical Research Saarland, Saarbruecken, Saarland 66123, Germany; Centre for Bioinformatics, Saarland University, Saarbruecken, Saarland 66123, Germany; Drug Bioinformatics, Helmholtz Institute for Pharmaceutical Research Saarland, Saarbruecken, Saarland 66123, Germany; Centre for Bioinformatics, Saarland University, Saarbruecken, Saarland 66123, Germany; Medical Faculty, Saarland University, Homburg, Saarland 66421, Germany

## Abstract

**Motivation:**

Enzymes are essential catalysts in many cellular processes. Understanding their interactions with small molecules, such as regulators, cofactors, and most importantly, substrates, is crucial for understanding the biochemical processes that occur in cells. Correctly interpreting the roles of small molecules that interact with enzymes is key to elucidating enzyme function. Recently, enzyme–small-molecule interaction prediction has attracted growing interest from computational methods, especially deep learning. As a result, researchers have published several datasets and numerous models with remarkable performance.

**Results:**

In this work, we critically examine one of the most popular datasets and four models trained on it, identifying similarity-induced information leakage that may overinflate reported model performance in out-of-distribution applications. We show that the inspected models are susceptible to information leakage, and their performance is not better than statistical baselines when the leakage is removed. Furthermore, controlling for leakage due to protein sequence similarity alone still yields high predictive performance, whereas performance decreases substantially when common substrates shared between training and test sets are removed from one of them, and approaches random prediction when structurally similar ligands are separated between splits. Thus, the investigated models generalize considerably better across enzyme sequence space than across ligand chemical space, indicating that their reported performance depends strongly on the small-molecule similarity between training and evaluation data.

**Availability and implementation:**

All data splits we calculated in this study are available on Zenodo (DOI: 10.5281/zenodo.18786394). The code is available on GitHub and backed up on Zenodo (DOI: 10.5281/zenodo.18788609).

## 1 Introduction

Enzymes are proteins that catalyze essential biological reactions, playing key roles in metabolism, energy transfer, signal transduction, and many other cellular processes (Berg *et al.* 2012). They do so by binding a small chemical molecule (substrate) and transforming it into another molecule (product). Many enzymes also require other (small) molecules, such as cofactors or coenzymes, for catalytic activity, while their activity can be modulated by inhibitors, activators, and other regulatory small molecules. For example, RuBisCO (Enzyme Commission, or EC, number 4.1.1.39) is involved in photosynthesis in plants and catalyzes the fixation of atmospheric carbon dioxide into glucose and other sugars (Andersson 2008). Understanding interactions between enzymes and small molecules is fundamental in biology and drug design.

Computational prediction of enzyme-small-molecule interactions is a subfield of the broader field of predicting protein–ligand interactions (PLIs). Generally, there are multiple ways to predict PLIs: (i) qualitatively, i.e. whether a pair of protein and ligand is binding or not, (ii) quantitatively, predicting how strong an interaction is measured as binding affinity, and (iii) positionally, where a docking algorithm determines the pose of the ligand relative to the protein (Trott and Olson 2010, Corso *et al.* 2022, Lacour *et al.* 2026). In the latter case, it is typically assumed that the ligand binds to the protein, as most docking algorithms always return a pose, while the first two types of predictors can also correctly predict noninteracting pairs. In this work, we focus on qualitative prediction methods for interactions between enzymes and small molecules.

Predicting enzyme–small-molecule interactions is challenging due to their inherent complexity caused by the high specificity of enzymes, dynamic and transient interactions with ligands, and conformational flexibility during catalysis (Hammes *et al.* 2011, Callender and Dyer 2015, Riziotis *et al.* 2022). Additionally, enzymes are often multifunctional and promiscuous binders (Khersonsky and Tawfik 2010, Kuo *et al.* 2016). The evolutionary diversity of enzymes and the lack of high-quality data exacerbate these challenges, making the design of efficient computational methods to predict enzyme functionality even more challenging (Hammes *et al.* 2011; Riziotis *et al.* 2022).

Training a model to predict all interaction types (e.g. substrates, regulators, cofactors, and noninteractors) remains challenging due to the lack of comprehensive, curated enzyme–small-molecule datasets; consequently, many studies reduce the task to a binary classification problem, distinguishing known, interacting enzyme–small-molecule pairs from either experimentally validated (Pertusi *et al.* 2017) or synthetically generated (Kroll *et al.* 2023, 2024, Cui *et al.* 2025, Du *et al.* 2025, Nie *et al.* 2025, Qian *et al.* 2025) negative samples, where interacting small molecules are primarily understood as substrates. Computational methods for predicting enzyme–substrate interactions have thrived recently, evidenced by the publication of several new models and datasets (Pertusi *et al.* 2017, Kroll *et al.* 2023, 2024, Du *et al.* 2025, Nie *et al.* 2025).

Because there is no systematically curated experimental dataset of noninteracting enzyme–small-molecule pairs, these pairs must be generated synthetically to train computational models. Kroll *et al.* introduced a new dataset combining experimentally validated interacting enzyme–small-molecule pairs and synthetically generated noninteracting pairs. After splitting the data into train and test sets, for each experimentally validated enzyme–substrate pair, Kroll *et al.* sampled three substrates from the same split that were structurally similar to the actual substrate. These three new pairs are assumed to be non-interacting (Kroll *et al.* 2023). Together with this dataset (hereafter called the *ESP dataset*), a deep learning-based model, enzyme-substrate prediction (ESP), has been presented to solve the posed classification task. This dataset has been used in various follow-up studies, including ProSmith, FusionESP, and OmniESI (Kroll *et al.* 2024, Du *et al.* 2025, Nie *et al.* 2025), which reported remarkable classification performance with AUROC values (area under the receiver operating characteristic curve) ranging from 0.956 to 0.972.

*Information leakage* is a phenomenon of unintended data sharing between training and test data of a model that can yield overly optimistic estimates of the model’s performance. Information leakage (also called *data leakage*) refers to the situation in which a model, during development, has access to information about the evaluation data that are not present in the inference data (Bernett *et al.* 2024b). Bernett *et al.* argue that information leakage is a multifaceted phenomenon. In particular, if the intended applications of a model are broad and poorly defined, sheer similarities between data points in the training and test data can lead to information leakage for *out-of-distribution* (OOD) applications.

In this work, we focus on the impact of these inter-sample similarities on model performance in ESP with an intended application to novel enzymes and drugs. Specifically, we focus on two aspects of inter-sample similarity: sequence similarity between enzymes and structural similarity between small-molecule ligands. For ligands, we assess structural similarity using Tanimoto similarity between Morgan fingerprints. Accordingly, the OOD settings considered in this study assess generalization to sequence-dissimilar enzymes, structurally dissimilar ligands, or both. These definitions represent the specific OOD scenarios investigated here; other notions of distributional shift, e.g. with respect to protein structure, ligand scaffolds, or binding mechanisms, are conceivable but outside the scope of this study.

However, there are different conceivable application scenarios, such as drug screening and drug repurposing, in which the inference samples can also come from the same data pool as the training data. For a broadly applicable model, these scenarios and different levels of dissimilarity between the training and test data need to be evaluated during model development to obtain reliable estimates of how well a model performs on so-called out-of-distribution data and, therefore, how well it generalizes. Thus, the concepts of information leakage and out-of-distribution are very much interconnected. In this work, we investigate the influence of similarity-induced information leakage on the performance of four state-of-the-art models and two baselines, and retrain and evaluate them in several out-of-distribution settings: ESP (Kroll *et al.* 2023), ProSmith (Kroll *et al.* 2024), FusionESP (Du *et al.* 2025), and OmniESI (Nie *et al.* 2025). All four models acknowledge the importance of information leakage and report performance drops if evaluated on molecules or drugs unseen in training. They consider two OOD settings: (i) small molecules in the test set that are scarcely or not at all represented in the training set and (ii) enzymes with 40%–60% and <40% sequence identity to enzymes in the training set. These are typical examples of so-called *inter-sample similarity-induced information leakage*. An important difference to note is that in their publication, the models have not been trained on OOD splits but only evaluated on OOD subsets of the test set of the ESP split. In this work, we retrained the models for each setting.

In our previous work (Joeres *et al.* 2025), we investigated in depth the problem of splitting protein–small-molecule interaction datasets to minimize information leakage and proposed a total of six OOD settings, of which the above-mentioned OOD tests correspond to only two. Hence, we set out to investigate the models by re-splitting the original ESP dataset using our recently published DataSAIL (Joeres *et al.* 2025) and demonstrate that performance declines significantly as intersample similarity-induced information leakage decreases further. We show that the effect is strongly asymmetric between the two data modalities: reducing protein sequence similarity between splits alone has a comparatively modest effect on model performance, whereas reducing ligand structural similarity causes a pronounced performance decline. When structurally similar ligands are separated between training and evaluation data, model performance approaches random levels.

## 2 Methods

In the ESP data-processing pipeline, experimentally validated interacting pairs of enzymes and small molecules are first clustered by enzyme similarity using CD-HIT (Li and Godzik 2006); the clusters are then randomly split into training and test sets. Importantly, no such clustering is conducted on the side of the small molecules. However, in ProSmith and FusionESP, the ESP dataset is initially divided into training, validation, and test sets. To ensure consistency, we recomputed this split to train all models. In the following, we refer to this method as the *ESP split* (Kroll *et al.* 2023). In addition to that split, we compute several splits generated by DataSAIL (Fig. 1): *protein-based* and *ligand-based one-dimensional splits* that split all interactions based on protein or ligand identifiers (I1${\;}_{L}$ and I1${\;}_{P}$, respectively) or similarity clusters (S1${\;}_{L}$ and S1${\;}_{P}$, respectively). These 1D splits relate to the scenarios of drug screening, i.e. testing a new drug against known ligands, I1${\;}_{L}$ and S1${\;}_{L}$, and drug repurposing, i.e. screening a new ligand against known drugs, I1${\;}_{P}$ and S1${\;}_{P}$ (Pushpakom *et al.* 2019, Wu *et al.* 2024). And lastly, a *2D split*, S2, that combines S1${\;}_{L}$ and S1${\;}_{P}$ splits, therefore minimizing protein and ligand similarities between splits simultaneously. Because the S2 split removes data, we computed an ESP${\;}_{S2}$ split following the ESP split algorithm, using only the data in the S2 split. This is done to compare the ESP split with the S2 split, the strictest DataSAIL split. In each splitting scheme, we split the data into 70% for training, 10% for validation and hyperparameter tuning, and 20% for testing (Table S1, available as supplementary data at *Bioinformatics* online). Here, we deviate from the original split ratios of Kroll *et al.* who used 80% for training and validation and 20% for testing (Kroll *et al.* 2023), to allow for hyperparameter tuning. This also explains the deviation of numbers on the ESP split in Table 1.

**Figure 1:**
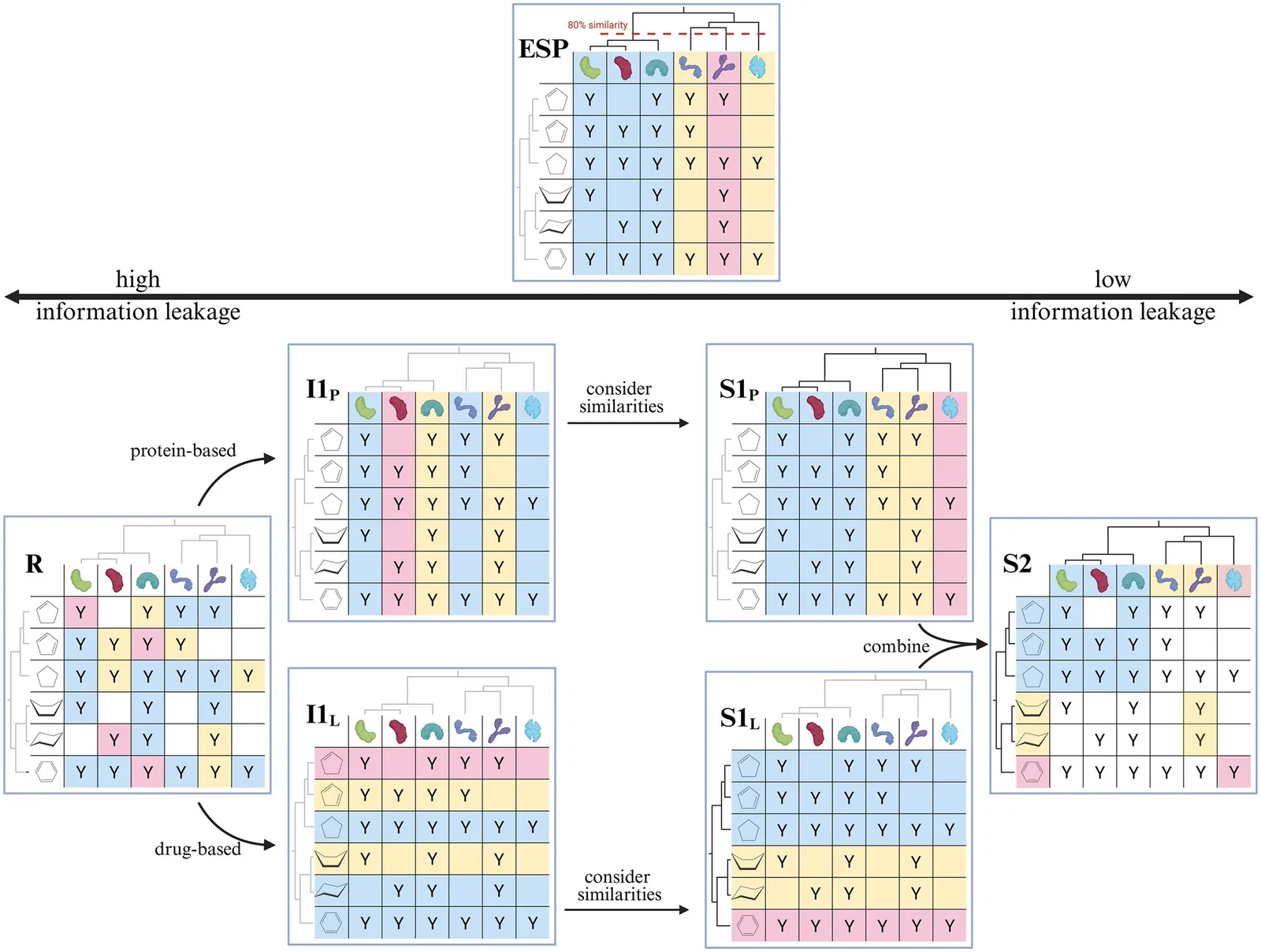
Visualization of data splitting techniques used in this study. The 2D matrix represents the dataset of experimentally verified enzyme–substrate pairs. The symbol ‘Y’ indicates the presence of a measurement, while the dendrograms next to the rows and columns indicate similarities between samples. Grey indicates techniques that do not use similarities, and black highlights techniques that do. The dashed red line marks the level beyond which connected nodes have a similarity of 80% or more. Training samples are highlighted in blue, and test samples are in red. Unassignable tiles are left white. In the ESP split, the test set comprises proteins with pairwise similarity to the training data below 80%. We compare this with the five splits below, where we split the dataset either by individual ligand (I1${\;}_{L}$), by individual protein (I1${\;}_{P}$), or by their clusters (S1${\;}_{L}$ and S1${\;}_{P}$). Those splits make sure that no two interactions from different splits share the same (or similar, in the case of S1 splits) ligands (or proteins). In these splits, the data are split only along one dimension (proteins or ligands); the S2 split combines the leakage prevention measures from S1${\;}_{L}$ and S1${\;}_{P}$ at the cost of not assigning all interactions, as some interactions will consist of two molecules in different splits (Joeres *et al.* 2025).

**Table 1:** Comparison of performance metrics reported in the original publications and the retrained models and statistical baselines. We computed the accuracies, the AUROCs, and MCCs on the test set after re-training the models (Ours) and compared them to the values reported (Rep.) in the compared work (Kroll et al. 2023, 2024, Du et al. 2025, Nie et al. 2025). The metrics in the columns for reported similarity-based cold-protein split (S1-Rep.) refer to the values from the original publications computed on the set of proteins with sequence identity to the training set. Similarly, the reported similarity-based 2D cold split (S2-Rep.) is the performance on the subset of interactions in the test set with unseen small molecules and enzyme sequence identity levels of 0%–40% to the training set. The best performance per metric and split is highlighted in bold.

	ESP		**ESP_S2_**		**I1_P_**		**S1_P_**		**I1_L_**		**S1_L_**		S2	
	Rep.	Ours	Rep.	Ours	Rep.	Ours	Rep.	Ours	Rep.	Ours	Rep.	Ours	Rep.	Ours
**Accuracy (** ↑ **)**														
‘Not interacting’ baseline	–	0.738	–	0.739	–	0.735	–	0.737	–	0.698	–	**0.735**	–	**0.733**
Configuration model	–	0.743	–	0.739	–	0.740	–	0.737	–	0.699	–	**0.735**	–	**0.733**
ESP (ESM-1b_ts_ + GNN)^a^	0.92	0.887	–	0.879	–	0.902	0.89	0.865	0.90	0.566	–	0.407	0.71	0.545
ProSmith	0.94	0.923	–	0.871	–	0.930	–	0.865	–	0.754	–	0.633	–	0.581
FusionESP	0.95	**0.933**	–	**0.916**	–	**0.943**	0.93^b^	**0.889**	0.81^b^	**0.760**	–	0.722	–	0.728
OmniESI	0.95	0.907	–	0.886	–	0.905	0.91	0.859	–	0.690	–	0.715	–	0.685
**AUROC (** ↑ **)**														
‘Not interacting’ baseline	–	0.500	–	0.500	–	0.500	–	0.500	–	0.500	–	0.500	–	0.500
Configuration model	–	0.574	–	0.583	–	0.590	–	0.574	–	0.501	–	0.499	–	0.500
ESP (ESM-1b_ts_ + GNN)^a^	0.96	0.930	–	0.921	–	0.937	0.93	0.887	0.71	0.565	–	0.548	0.59	0.516
ProSmith	0.97	0.954	–	0.892	–	0.957	–	0.889	–	**0.792**	–	0.555	–	0.530
FusionESP	0.96	**0.955**	–	**0.944**	–	**0.968**	0.95^b^	**0.912**	0.69^b^	0.724	–	0.550	–	0.543
OmniESI	0.97	0.944	–	0.932	–	0.946	0.95	0.876	–	0.587	–	**0.561**	–	**0.545**
**MCC (** ↑ **)**														
‘Not interacting’ baseline	–	0.000	–	0.000	–	0.000	–	0.000	–	0.000	–	0.000	–	0.000
Configuration model	–	0.140	–	0.111	–	0.142	–	0.121	–	0.000	–	0.000	–	0.000
ESP (ESM-1b_ts_ + GNN)^a^	0.78	0.711	–	0.691	–	0.757	0.72	0.461	0.27	0.104	–	0.030	0.15	0.022
ProSmith	0.85	0.796	–	0.652	–	0.818	0.78	0.645	0.29	0.352	–	0.064	–	0.025
FusionESP	0.86	**0.824**	–	**0.776**	–	**0.852**	0.82^b^	**0.704**	0.30^b^	**0.375**	–	0.020	–	0.004
OmniESI	0.86	0.758	–	0.694	–	0.756	0.76	0.624	–	0.129	–	**0.070**	–	**0.066**

a Except for the reported ESP models, which were trained using cross-validation (train-test split), all other models were trained using a standard train-validation-test split.

b Values for FusionESP-XL with ESM-2-3B & MolFormer, as the paper only reports these values for this model.

To compute the clusters for the cluster-based splits, we use CD-HIT (Li and Godzik 2006) for the enzymes and the following procedure for the small molecules: we compute Morgan fingerprints for each molecule, then all pairwise Tanimoto similarities thereof, and lastly we use spectral clustering to find clusters within the set of small molecules.

Theoretically, DataSAIL’s cluster-based splits, S1${\;}_{L}$, S1${\;}_{P}$, and S2, are the hardest to learn because similarity-based splits remove information leakage more rigorously. We have also demonstrated this for several drug–target interaction datasets (Joeres *et al.* 2025), but not for the ESP dataset. The ESP split and the CD-HIT-based S1${\;}_{P}$ split of this study are conceptually similar because both use CD-HIT to reduce protein overlap between splits. However, in the S1${\;}_{P}$ split, DataSAIL assigns whole CD-HIT clusters to the training, validation, and test sets while satisfying the specified split-size and stratification constraints. The ESP split only removes samples from the test set that have more than 80% sequence identity to any sequence in the training set. Therefore, DataSAIL’s S1${\;}_{P}$ split may have higher individual leakage but lower total leakage, as evidenced by Table S2, available as supplementary data at *Bioinformatics* online.

Information leakage can be quantified following Joeres *et al.* (2025) for each pair of splits *s* and $s^{\prime }$, e.g. training and test, of a dataset D:


(1) $${\text{Leakage}}_{s,s^{\prime }}=\frac{\sum_{u\in s,v\in s^{\prime }}w(u,v)}{\sum_{(u,v)\in \left(\begin{array}{c} D\\ 2 \end{array}\right)}w(u,v)},$$

where *u* and *v* are data points in D, which can be enzymes, substrates, or interactions thereof, and w:D×D→R is a similarity function between data points in D. The numerator measures the similarity between distinct splits, while the denominator normalizes by total similarity in the dataset. Lower leakage values indicate greater dissimilarity between the corresponding splits, resulting in a larger distributional shift between them.

In our analysis, we not only compare the four models mentioned but also two statistical baselines: (i) the ‘not interacting’ baseline, which always predicts ‘not interacting’ and (ii) the configuration model, making its predictions based on the node degrees (Chatterjee *et al.* 2023). An overview of the model and baseline architectures and details of the training process can be found in the Supplementary Information.

## 3 Results

We retrained the four models—ESP (Kroll *et al.* 2023), ProSmith (Kroll *et al.* 2024), FusionESP (Du *et al.* 2025), and OmniESI (Nie *et al.* 2025)—on the splits described above and performed hyperparameter tuning on the validation sets. All performance metrics calculated here are extracted from the same test sets for all models. The ESP column of Table 1 shows that our study reproduced the results from the original papers within a minor error margin.

To investigate data leakage in the ESP dataset and assess the models’ generalizability to new enzymes and ligands, we applied various splitting methods using DataSAIL. The leakage was quantified according to the similarity-based metric defined in Equation (1). For small molecules, pairwise similarities were calculated from 2048-bit Morgan fingerprints (radius = 2) using the Tanimoto coefficient. Enzyme sequence similarities were calculated using DIAMOND (Buchfink *et al.* 2015). The resulting similarity matrices were used to quantify similarities between the training, validation, and test sets. First, we compared molecule-similarity leakage (MSL) and protein-similarity leakage for different protein-based and ligand-based splits, with information leakage calculated using similarity matrices on substrates and enzymes, respectively (Table S2, available as supplementary data at *Bioinformatics* online). We observed that splitting a protein–ligand dataset based solely on the protein or the ligands reduces leakage in that axis, but the leakage along the other axis remains high. This can also be observed from the total-similarity leakage, which is computed as MSL+PSL. Overall, ligand-based splits were more effective at minimizing total data leakage, with the S1${\;}_{L}$ split achieving the lowest leakage among the 1D split methods. The lowest overall data leakage was observed in the S2 split, which splits data based on data point similarity along both axes (Fig. 2). The reason why protein-based splits consistently show higher data leakage than molecule-based splits can be the imbalance in the ESP dataset, which includes 12 010 unique proteins and only 1354 unique small molecules, creating inherent challenges for splitting the data. The enzyme–ligand interaction datasets can be formally represented as a bipartite graph where enzymes and substrates form two disjoint node sets, with edges representing observed interactions. When applying protein-based splitting to this structure, the high connectivity of substrates across enzyme partners frequently creates bridges between training and test partitions, as many substrates interact with multiple enzymes. Their inclusion in the training and test sets across different enzyme partners introduces unavoidable leakage. This architectural constraint explains why protein-based splits consistently show higher data leakage than molecule-based splits in our experiments.

**Figure 2:**
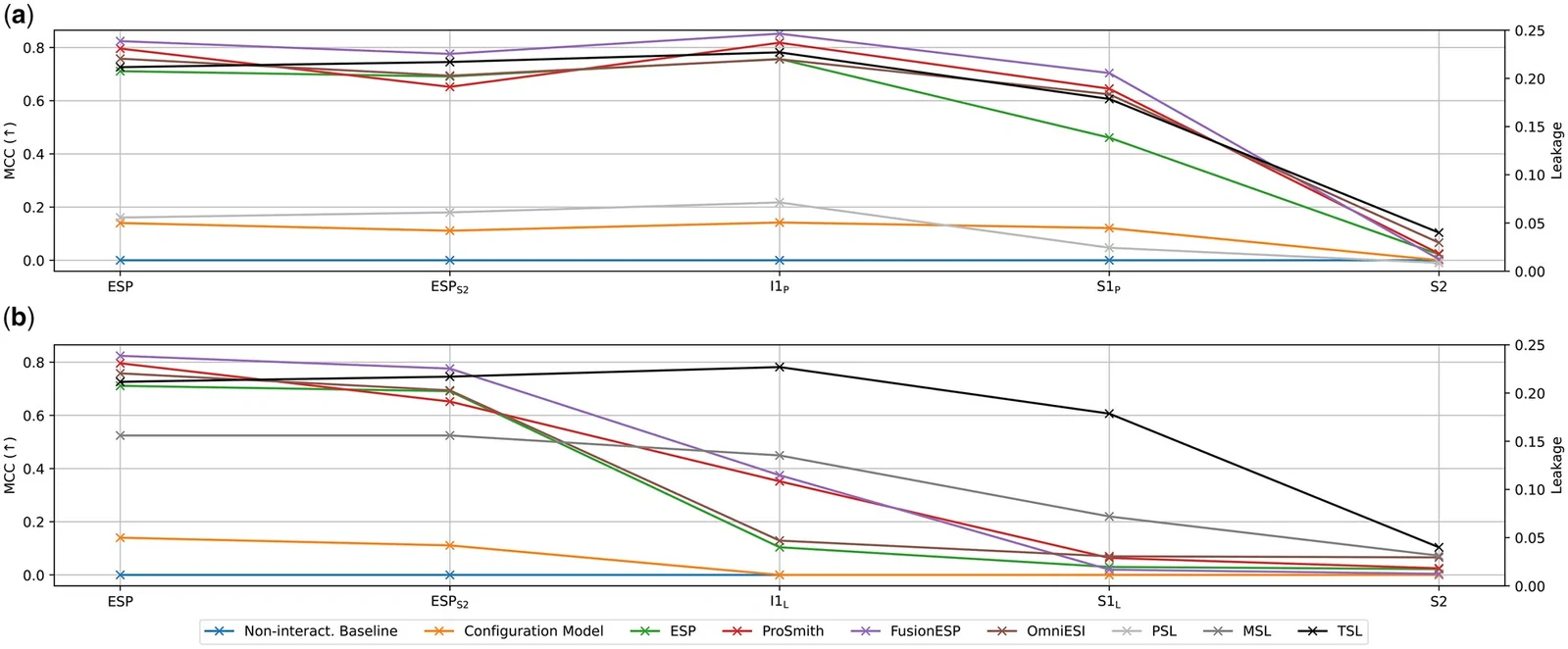
Models’ performance as a function of decreasing information leakage. (a) Models’ performance and total- and protein-similarity leakage (TSL and PSL), as they decrease in different protein-based splits. (b) Models’ performance, total and MSL across different small molecule-based splits.

In the development of the models on the ESP split, information leakage was controlled only along the protein axis, ensuring that no protein in the validation or test sets had more than 80% sequence identity to any protein in the training set. In addition to evaluating the models on the full ESP split test set, the authors also evaluated them on subsets thereof. Two of these subsets are all proteins with 0%–40% sequence identity to any sequence in the training set (S1${\;}_{P}$-Rep.), and another one contains only small molecules that were not present in the training set (I1${\;}_{L}$-Rep.). It is important to note that, in the original publications, the model was not retrained for these evaluation settings, whereas we trained a separate model for each splitting technique.

This trend is also reflected in the decreasing performance as splits leak less information and thus become more difficult to memorize (see Table S2, available as supplementary data at *Bioinformatics* online, the *r*-columns on the right). The Pearson correlation coefficients were calculated between the similarity leakage rows in the upper part of Table S2, available as supplementary data at *Bioinformatics* online, and the area under the receiver operating characteristic curve (AUROC) values in the lower part. Previous studies showed that FusionESP performed best among the three models, with ProSmith as the second-best model (Du *et al.* 2025). Interestingly, in our experiments for the I1${\;}_{L}$ split, ProSmith performs best, and for the S1${\;}_{L}$ split and the S2 split, all models perform almost as a random guess (Table 1 and Fig. 2).

Figure 2 visualizes how performance decreases as data splits become increasingly out-of-distribution and shows that encoding the small molecules with embeddings (ProSmith, FusionESP) instead of fingerprints or graphs (ESP, OmniESI) enables better generalization to similar ligands (ESP to I1${\;}_{L}$). However, both options generalize worse to out-of-distribution data than protein encoders. The performance only drops to random levels when small-molecule leakage is removed (Fig. 2A, between splits S1${\;}_{P}$ and S2). For small-molecule-based splits (Fig. 2B), this is even clearer: the performance drop from the ESP splits to I1${\;}_{L}$ and S1${\;}_{L}$ shows that the leakage along the protein axis does not help the model.

We observe a similar pattern across the reported Matthews correlation coefficients (MCCs) from the three publications, recalculated MCCs from retrained models, and MCCs on leakage-reduced splits (Table 1): a decrease in MCC correlates with reduced information leakage. Here, we loosely clustered the reported OOD splits with the most similar DataSAIL splits [e.g. ‘unseen small molecules’ (Kroll *et al.* 2023) for ESP with S1${\;}_{L}$ and ‘small molecules not represented in the training set and an enzyme sequence identity level of 0%–40% to the training data’ (Kroll *et al.* 2023) with S2]. The differences in the MCC values for these OOD splits can be explained by the procedural differences in the algorithms used to create the OOD sets and the DataSAIL splits. While in the original publications, the authors extracted the OOD subsets from the ESP split test set, we compute splits for each OOD setting individually. This increases the size of the OOD test set but also results in different training and validation sets each time. Additionally, the reported equivalent to our S1${\;}_{P}$ split contains all enzymes with <40% sequence identity to any enzyme in the training set. DataSAIL does not provide a fixed upper bound on sequence identity, but the S1${\;}_{P}$ split minimizes the total similarity between the training, validation, and test enzyme sequences.

## 4 Discussion

In this study, we highlight crucial deficiencies in the accepted procedures for training models for enzyme–small-molecule interaction prediction that were underexplored before. Existing models are very good at predicting interactions between molecules they encountered during training but struggle to generalize to unseen or OOD data. While generalization to unseen enzymes, even significantly different ones, is good—e.g. FusionESP achieves an AUROC of 0.912 and an MCC of 0.704 on a S1${\;}_{P}$ split—generalization to new small molecules remains poor. This also translates to settings where both the enzyme and the small molecule are new at evaluation time (i.e. S2 split).

Our analysis also fills gaps in previous evaluations (Table 1). It has not previously been investigated how the models perform when the small molecules are structurally dissimilar rather than merely different SMILES strings. Previously, any nonidentical molecules have been considered OOD. Here, we extend this to a more fine-grained notion of molecular similarity and show that models cannot generalize significantly beyond near-identical molecules.

These findings also align well with other reports on data leakage in deep learning-based methods for protein–protein interaction prediction (Bernett *et al.* 2024a; Reim *et al.* 2025). The group showed across multiple works that state-of-the-art deep learning models for protein–protein interaction prediction do not achieve accuracy above 0.65 when information leakage is rigorously removed. Table 1 shows similar results for ESP models. For the S1${\;}_{L}$ and S2 splits, the deep learning models achieve performance below the statistical baselines (see Table 1 and Fig. 2). Other than in Chatterjee *et al.* (2023), the configuration model operating on the degree composition of the dataset does not provide a strong statistical baseline, because the nodes in the bipartite interaction graph have almost identical degrees due to the way negative data was generated (see Fig. S2, available as supplementary data at *Bioinformatics* online).

Another aspect worth investigating is the process to generate negative data. Kroll *et al.* generate the negative data after splitting the positive interactions into training and test sets. In that step, the negative data is generated by selecting the three most similar small molecules from the whole set of small molecules, not just those already present in a split (Kroll *et al.* 2023). Therefore, this procedure may amplify the small-molecule-induced information leakage already present in the ESP split. Additionally, taking the three most similar and adding them as noninteracting contradicts the general assumption in the field that structurally similar molecules have similar interactions.

## Supplementary Material

btag733_Supplementary_Data

## Data and availability

All data splits we calculated in this study are available on Zenodo (DOI: 10.5281/zenodo.18786394). The code is available on GitHub and backed up on Zenodo (DOI: 10.5281/zenodo.18788609).
