# Supplementary material for: Information leakage in enzyme-substrate prediction
Source: Bioinformatics. 2026 Oct 1;42(10):btag733. doi: 10.1093/bioinformatics/btag733 (PMC13668884; doi:10.1093/bioinformatics/btag733)
Supplement: Supplementary file 1 [file btag733_supplementary_data.pdf]

Information Leakage in Enzyme Substrate Prediction

Supplementary Material

Vahid Atabaighi Elmi[1,2 * †], Roman Joeres[1,2 †], and Olga V. Kalinina[‡1,2,3]

[1]Drug Bioinformatics, Helmholtz Institute for Pharmaceutical Research Saarland, 66123 Saarbruecken, Germany
[2]Center for Bioinformatics, Saarland University, 66123 Saarbruecken, Germany
[3]Medical Faculty, University Hospital Saarland, 66421 Homburg, Germany

1 Overview of the analyzed models

Enzyme Substrate Prediction model (ESP) (Kroll et al., 2023). The ESP model follows the classical architecture of protein-ligand interaction prediction models, with independent encoders for the enzyme and small-molecule components, whose embeddings are concatenated and fed into a prediction head. The enzyme encoder is a modified ESM-1b model (Rives et al., 2021) in which the authors added an additional 1280-dimensional token representing the entire enzyme, learning a task-specific representation of the enzyme. Small molecules are either represented as 1024-dimensional ECFP descriptors with a radius of 3 or using a graph neural network. The prediction head is a gradient-boosting model (Figure 1a). The authors report very good performance in testing (AUC: 0.956), but observed a significant decline in performance when evaluating only the out-of-distribution parts of the test set (AUC: 0.71; MCC: 0.27; Table 1).

PROtein-Small Molecule InTeraction, Holistic model (ProSmith) (Kroll et al., 2024). This model processes token-level ESM-1b protein embeddings and ChemBERTa2 (Ahmad et al., 2022) ligand embeddings in a multimodal transformer. Both embeddings are concatenated with a 0-vector as a separator in between and prepended with a unit vector as a CLS token. From this transformer, the CLS-token embedding is extracted and paired with the mean-pooled embeddings from ESM-1b and ChemBERTa2. The CLS token alone, the combined ESM-1b and ChemBERTa2 embeddings, or their combination with the CLS token are then used as input to a gradient-boosting prediction head. In this study, we focus on the model trained using the combination of all three representations (Figure 1b). ProSmith is pre-trained on IC50 values from the BindingDB dataset (Liu et al., 2007), and then task-specifically fine-tuned on the downstream datasets Davis (Davis et al., 2011) and ESP. To ensure that the downstream validation and test splits are truly OOD, all proteins and ligands from those splits were removed from the pre-training data in BindingDB. Like ESP, ProSmith was separately evaluated on the OOD parts of the test set and showed clear improvement over ESP models to an MCC of 0.29 (Table 1).

*Current Affiliation: Department of Chemistry and Molecular Biology, University of Gothenburg and Wallenberg Centre for Molecular and Translational Medicine, University of Gothenburg

†V.A.E and R.J. contributed equally to this work.

‡Corresponding author: olga.kalinina@helmholtz-hips.de

FusionESP (Du et al., 2025). FusionESP also follows the general idea of having two independent encoders for proteins and molecules. It updates the previously used ESM-1b protein encoder to an ESM2 encoder (Lin et al., 2023) and substitutes ChemBERTa2 with MoLFormer (Wu et al., 2023). In this study, we focus on the FusionESM model with the ESM2-650M model over the variant utilizing the ESM2-3B model. On the forward pass, the mean-pooled embeddings are separately passed through a projection head comprising two streams, each with two rounds of a dense layer, batch normalization, and either ReLU activation or L2 regularization. The prediction is then calculated as the cosine similarity between the two resulting 128-dimensional embeddings (Figure 1c). This similarity is then optimized using a mean-squared error loss function. This has the effect that the embeddings of proteins and ligands lie in the same latent space, and that the embeddings of binding molecules are close to each other, while those of non-binding pairs are pushed apart. Furthermore, embeddings of proteins with similar binding patterns are projected close together. The evaluation shows that FusionESP both outperforms ESP and ProSmith, with the gap to ProSmith smaller than to ESP. For OOD subsets of the test data, FusionESP's margin over ProSmith further increases to AUC 0.8185 (see Table 1).

OmniESI (Nie et al., 2025). Similar to the previous three models, OmniESI has two separate input streams for the enzyme and the small molecules. The enzymes are encoded using the pretrained ESM-2 650M model (Lin et al., 2023), while the small molecules are represented as 2D graphs and fed into a trained GCN encoder (Kipf & Welling, 2016). These separate encodings are processed by a first conditional network where the enzyme representation serves as a condition for further knowledge extraction of the substrate and vice versa. Therefore, the resulting representations contain enzymatic reaction information. In a second conditional network, these representations are further enriched and fine-tuned to yield a catalysis-focused final representation from which the binary prediction can be made (Figure 1d). Additionally, OmniESI can adapt to other downstream tasks such as kinetic parameter or mutational effect prediction. In the publication, OmniESI shows improved performance over ESP in protein OOD settings with an MCC of 0.76 (see Table 1).

"not interacting" Baseline. This baseline always predicts the majority class from the training data, ignoring any information about the enzyme or small molecule. In all splits, the majority class is always the negative, "not interacting" class, hence the name.

Configuration Model. The AI-Bind paper introduced a baseline making predictions based on the similarity of node degrees to the training set. For the full mathematical description, we refer the reader to Chatterjee et al. (Chatterjee et al., 2023). We reproduced this baseline using the bcim Python package. Since the dataset contains only positive experimental interactions and all negatives were generated synthetically and uniformly equal for all proteins, the node degrees are significantly different from those in natural protein-ligand interaction datasets. Hence, the Configuration Model often reduces to the "not interacting" baseline, not yielding a competitive baseline for most settings.

All models were retrained using Python 3.12 and PyTorch 2.8 on a single workstation with an NVIDIA RTX 3090 GPU with 24GB RAM and 16 CPUs with 128GB RAM. The data were downloaded from the ESP GitHub repository and the linked Zenodo archive (Kroll et al., 2023). For ProSmith, FusionESP, and OmniESI, we also followed the instructions from their respective GitHub repositories (Kroll et al., 2024; Du et al., 2025; Nie et al., 2025).

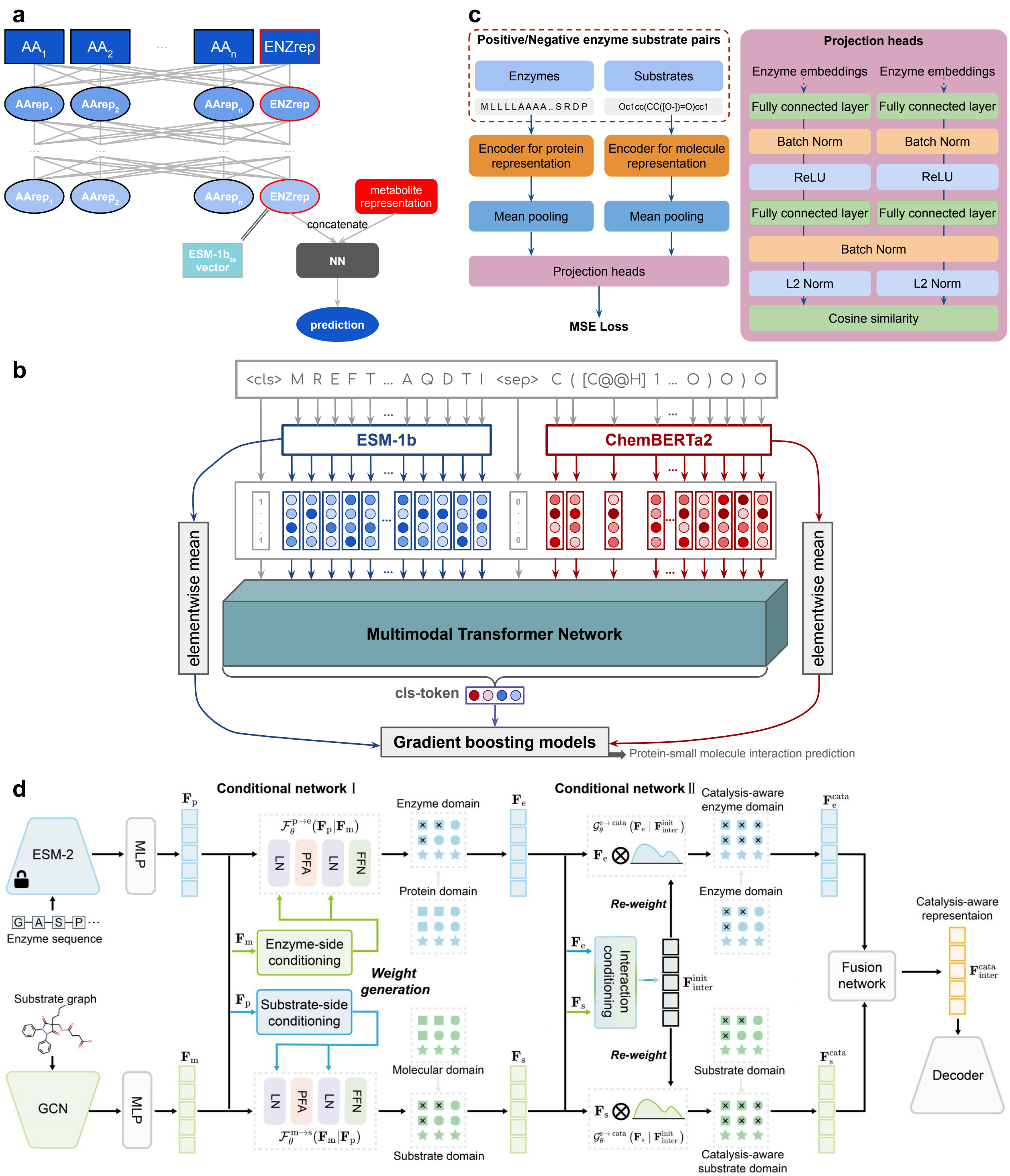

Figure 1: Caption

Table 1: Overview of different split strategies with corresponding sample ratios and negative-to-positive (Neg./Pos.) data ratios.

	Training				Validation				Test			
	total	# enz	# lig	PA	total	# enz	# lig	PA	total	# enz	# lig	PA
$I1_L$	48 938	8 986	940	0.265	6 966	1 709	159	0.266	12 121	3 495	255	0.365
$S1_L$	48 489	9 558	871	0.267	6 825	1 611	152	0.273	13 839	3 139	331	0.268
$I1_P$	48 660	8 012	1 206	0.265	6 982	1 147	486	0.263	13 844	2 851	696	0.267
$S1_P$	48 497	8 330	1 131	0.267	6 941	1 281	378	0.268	13 919	2 399	632	0.266
ESP	48 541	8 420	1 197	0.267	7 008	1 176	462	0.265	14 034	2 409	682	0.264
S2	22 128	4 622	638	0.265	3 095	640	231	0.258	6 344	1 291	360	0.263
ESP_{S2}	25 604	5 334	422	0.270	1 837	417	155	0.273	3 622	806	179	0.271

Table 2: **Data leakage and model performances.** Comparison of leakages between different subsets, the AUROC of the tested models, and the Pearson correlation coefficient r between AUC-ROC and the leakage of the data splits. In bold, we highlighted the lowest leakage values per row, the best model performance per split, and the highest correlation between AUROC and total similarity leakage (TSL).

		ESP	ESP_{S2}	$I1_P$	$S1_P$	$I1_L$	$S1_L$	S2	r_{ESP}	r_{PS}	r_{FESP}	$r_{OmniESI}$
Leakage Source												
Train-Val	MSL	0.142	0.141	0.141	0.144	0.128	0.062	**0.023**	0.815	0.957	0.918	0.808
Train-Val	PSL	0.052	0.056	0.053	0.022	0.070	0.058	**0.009**	0.099	0.323	0.221	0.109
Train-Val	TSL	0.194	0.197	0.194	0.166	0.198	0.120	**0.032**	0.675	**0.866**	0.800	0.674
Train-Test	MSL	0.284	0.285	0.284	0.278	0.249	0.136	**0.067**	0.837	0.966	0.933	0.833
Train-Test	PSL	0.100	0.112	0.133	0.044	0.089	0.112	**0.015**	0.409	0.459	0.428	0.418
Train-Test	TSL	0.384	0.397	0.417	0.322	0.338	0.248	**0.082**	0.783	**0.899**	0.863	0.783
Val-Test	MSL	0.042	0.042	0.042	0.041	0.029	0.018	**0.004**	0.908	0.963	0.958	0.901
Val-Test	PSL	0.015	0.015	0.028	0.007	0.016	0.012	**0.002**	0.494	0.623	0.567	0.507
Val-Test	TSL	0.057	0.057	0.070	0.048	0.045	0.030	**0.006**	0.840	**0.930**	0.904	0.840
AUC-ROC												
ESP (ESM-$1b_{ts}$ + GNN)		0.930	0.921	0.937	0.887	0.565	0.548	0.516	1.000	–	–	–
ProSmith		**0.954**	0.892	0.957	0.889	**0.792**	0.555	0.530	-	1.000	–	–
FusionESP		0.955	**0.944**	**0.968**	**0.912**	0.724	0.550	0.543	–	–	1.000	–
OmniESI		0.944	0.932	0.946	0.876	0.587	**0.561**	**0.545**	–	–	–	1.000

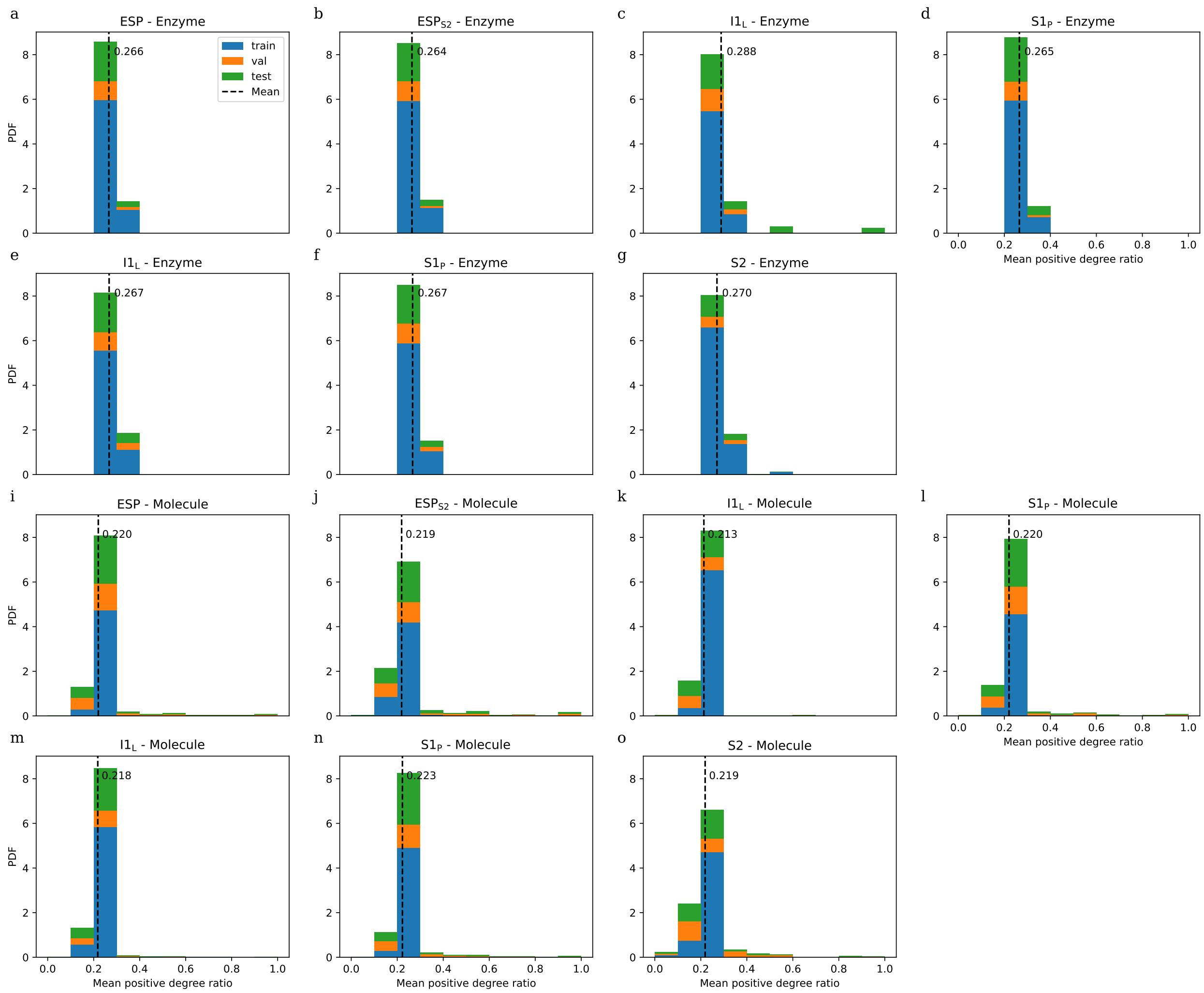

Figure 2: **Degree ratios for each split.** Following the example of the AI-Bind paper, we compute the positive-to-total annotation ratio for enzymes and small molecules in each split. We can see that they do not differ much between splits and within the training, validation, and test folds of individual splitting techniques. Unlike the BindingDB in AI-Bind, the negative data in the ESP dataset are synthetically generated; therefore, the node degrees do not follow standard degree distributions from natural protein-ligand interaction datasets. This also explains why the Configuration Model baseline is only slightly better than the "not interacting" baseline.

References

Ahmad W *et al.* Chemberta-2: Towards chemical foundation models. *preprint* 2022;DOI: 10.48550/arXiv.2209.01712.

Chatterjee A *et al.* Improving the generalizability of protein-ligand binding predictions with AI-Bind. *Nature Communications* 2023;**14(1)**;1989.

Davis MI *et al.* Comprehensive analysis of kinase inhibitor selectivity. *Nature Biotechnology* 2011;**29(11)**;1046-51.

Du Z *et al.* FusionESP: Improved Enzyme–Substrate Pair Prediction by Fusing Protein and Chemical Knowledge. *Journal of Chemical Information and Modeling* 2025;**65(6)**;2806-17.

Kipf TN, Welling M. Semi-supervised classification with graph convolutional networks. *preprint* 2016;DOI: 10.48550/arXiv.1609.02907.

Kroll A *et al.* A general model to predict small molecule substrates of enzymes based on machine and deep learning. *Nature Communications* 2023;**14(1)**;2787.

Kroll A, Ranjan S, Lercher MJ. A multimodal Transformer Network for protein-small molecule interactions enhances predictions of kinase inhibition and enzyme-substrate relationships. *PLOS Computational Biology* 2024;**20(5)**;e1012100.

Lin Z *et al.* Evolutionary-scale prediction of atomic-level protein structure with a language model. *Science* 2023;**379(6637)**;1123-30.

Liu T *et al.* BindingDB: a web-accessible database of experimentally determined protein–ligand binding affinities. *Nucleic Acids Research* 2007;**35(suppl_1)**;D198-D201.

Nie Z *et al.* OmniESI: A unified framework for enzyme-substrate interaction prediction with progressive conditional deep learning. *preprint* 2025;DOI: 10.48550/arXiv.2506.17963.

Rives A *et al.* Biological structure and function emerge from scaling unsupervised learning to 250 million protein sequences. *Proceedings of the National Academy of Sciences* 2021;**118(15)**;e2016239118.

Wu F, Radev D, Li SZ. Molformer: Motif-based transformer on 3d heterogeneous molecular graphs. *Proceedings of the AAAI Conference on Artificial Intelligence* 2023;**37(4)**;5312-20.
